# Adjuvant effect of enterotoxigenic *Escherichia coli* (ETEC) double-mutant heat-labile toxin (dmLT) on systemic immunogenicity induced by the CFA/I/II/IV MEFA ETEC vaccine: Dose-related enhancement of antibody responses to seven ETEC adhesins (CFA/I, CS1-CS6)

**DOI:** 10.1080/21645515.2019.1649555

**Published:** 2019-08-23

**Authors:** Hyesuk Seo, Ti Lu, Sachin Mani, A. Louis Bourgeois, Richard Walker, David A. Sack, Weiping Zhang

**Affiliations:** a Diagnostic Medicine/Pathobiology Department, Kansas State University College of Veterinary Medicine, Manhattan, KS, USA; b Department of Pathobiology, University of Illinois at Urbana-Champaign, Illinois, Il, USA; c PATH, Center for Vaccine Innovation and Access, Washington, DC, USA; d Department of International Health, Johns Hopkins University Bloomberg School of Public Health, Baltimore, MD, USA

**Keywords:** Dmlt, adjuvant, dose effect, CFA/I/II/IV MEFA, enterotoxigenic *Escherichia coli* (ETEC), antibody response

## Abstract

Double-mutant heat-labile toxin (dmLT, LT_R192G/L211A_) of enterotoxigenic *Escherichia coli* (ETEC) is an effective mucosal adjuvant. Recent studies have shown that dmLT also exhibits adjuvanticity for antigens administered parenterally. In this study, we subcutaneously (SC) immunized mice with the ETEC adhesin-based vaccine, CFA/I/II/IV MEFA (multiepitope fusion antigen), adjuvanted with dmLT and examined the impact of dmLT on antibody responses specific to the seven adhesins in the vaccine construction [CFA/I, CFA/II (CS1, CS2, CS3) and CFA/IV (CS4, CS5, CS6)]. Mice were immunized with a fixed dose of CFA/I/II/IV MEFA and ascending doses of dmLT adjuvant (0, 0.05, 0.1, 0.5 or 1.0 µg) to assess the potential dmLT dose response relationship. Data showed that dmLT enhanced systemic antibody responses to all seven antigens (CFA/I, CS1-CS6) targeted by MEFA in a dose-dependent way. The adjuvant effect of dmLT on the MEFA construct plateaued at a dose of 0.1 µg. Results also indicated that dmLT is an effective parenteral adjuvant when given by the SC route with the ETEC adhesin MEFA vaccine and that antibody enhancement was achieved with relatively low doses. These observations suggest the potential usefulness of dmLT for parenteral ETEC vaccine candidates and also perhaps for vaccines against other pathogens.

## Introduction

ADP-ribosylating bacterial toxins, heat-labile toxin (LT) of enterotoxigenic *Escherichia coli* (ETEC) and cholera toxin (CT) of *Vibrio cholerae*, are effective adjuvants for mucosal vaccines.^1–4^ LT (and CT) is a typical bacterial AB_5_ holotoxin, consisting of a ribosylating A subunit and five B subunits which form a pentameric ring to bind to host GM gangliosides receptors.^5^ The immunoregulatory mechanisms of LT (or CT) and derivatives, however, are not fully understood. It is thought that LT binding to host GM receptors facilitates antigen uptake across mucosal membranes and likely gains access to follicle-associated epithelium and the Peyer’s patches, thus up-immunoregulating antigens and enhancing (as an adjuvant) stimulation of antigen-specific immunity.^6,7^

While potent toxicity prevents the use of native LT as a safe adjuvant, LT mutants that have reduced toxicity but maintain adjuvant activity are considered the second generation of adjuvants.^8–13^ The remaining enterotoxicity in LT toxoids, however, was associated with Bell’s palsy when administrated intranasally in clinical trials.^10^ Further detoxification of LT mutant LT_R192G_ (mLT) led to a double-mutant LT, dmLT (LT_R192G/L211A_).^14^ While enzymatic activity for induction of intracellular cyclic GMP was further reduced, dmLT was found to retain LT adjuvanticity and was demonstrated to immunoregulate antigen-specific mucosal immune responses in oral, intragastric, sublingual and intranasal immunization studies.^14–21^

Recent studies suggested that dmLT adjuvant might have positive immunoregulatory properties when given parenterally with vaccine antigens.^22,23^ The potential impact of adding dmLT to a parenteral subunit ETEC vaccine, however, has not been fully investigated. In this study, we subcutaneously (SC) immunized mice with ETEC adhesin MEFA (multiepitope fusion antigen) CFA/I/II/IV, a leading and novel subunit ETEC vaccine candidate, and examined dmLT adjuvant activity in up-regulating antibody responses to the seven ETEC adhesin antigens included in the vaccine construct, CFA/I, CFA/II (CS1, CS2, CS3) and CFA/IV (CS4, CS5, CS6). Moreover, mice were immunized with CFA/I/II/IV MEFA supplemented with ascending doses of dmLT to determine if it could help to improve the anti-adhesin antibody responses in a dose-dependent manner.

## Materials and methods

### Antigen and adjuvant used in mouse immunization

Tag-less CFA/I/II/IV MEFA, which carries neutralizing epitopes of the major subunits of seven ETEC adhesins [CFA/I, CFA/II (CS1, CS2, CS3), CFA/IV (CS4, CS5, CS6)], was the antigen for mouse immunization. PATH provided the dmLT (LT_R192G/L211A_) used as the adjuvant.^24^

### Mouse subcutaneous immunization

A total of 60 eight-week-old female BALB/c mice (Charles River Laboratories International, Inc., Wilmington, MA), divided into six groups (10 mice per group), was included for immunization. Five groups of mice were each SC administered with 16 µg of CFA/I/II/IV MEFA protein and 0, 0.05, 0.1, 0.5, or 1 µg dmLT adjuvant accordingly, to examine potential role of adjuvant dose sparing. Mice received two booster injections at two-week intervals with the same dose as the primary. A group of ten mice without immunization served as the control. Blood samples were collected from each mouse prior to immunization and 14 days after final immunization. Mouse serum samples were stored at −20°C until use. The mouse immunization study was performed in accordance with the Animal Welfare Act by following the 1996 National Research Council guidelines, and the protocol was approved by the Kansas State University Institutional Animal Care and Use Committee.

### Mouse anti-CFA and anti-LT IgG antibody titration

Mouse serum anti-CFA adhesin and anti-LT IgG antibody titers were measured in ELISAs as previously described.^23–25^ Briefly, wells of 2HB plates (Thermo Scientific, Rochester, NY) coated with 100 ng of heat-extracted fimbriae (CFA/I, CS1, CS2, CS3, CS4, CS5), recombinant CS6 subunit protein CssA, or cholera toxin (CT; Sigma, St. Louis, MO) were incubated with two-fold diluted mouse serum samples (from 1:200 to 1:51200) for 1h at 37°C. Horseradish peroxidase (HRP)-conjugated goat anti-mouse IgG (1:3000; Bethyl Laboratories, Montgomery, TX) and 3,3ʹ,5,5ʹ-tetramethylbenzidine (TMB) Microwell Peroxidase Substrate System (KPL, Gaithersburg, MD) were used to measure optical density (OD_650_). IgG titers were calculated by the highest serum dilution producing an OD of 0.3 above the mean of the background and were presented in log_10_. Mouse serum or fecal IgA antibody responses were not included in this study due to the low and variable titers from the initial analysis.

### Mouse serum anti-LT antibody neutralization assay

Mouse serum samples were examined for anti-LT antibody neutralizing activity against CT toxin using cAMP EIA kit (Enzo Life, Farmingdale, NY) as previously described.^26,27^ Monolayer T-84 cells (ATCC, #CCL-248) were incubated with mouse serum sample pooled from each group (15 µl) pre-exposed to 10 ng CT (Sigma) in a 5% CO_2_ incubator for three hours. Cells were rinsed with 1x phosphate-buffered saline (PBS), lysed with 0.1 M HCl containing 0.5% Triton X-100 (Sigma, St. Louis, MO), and measured for intracellular cAMP levels (pmole/ml) by following the manufacturer’s protocol.

### Mouse serum antibody bacterial adherence inhibition assay

Mouse serum samples were examined for antibody inhibition activity against bacterial adherence to Caco-2 cells (ATCC, #HTB-37^TM^) as described previously.^24,25^ Briefly, 5 × 10^6^ CFU ETEC bacteria expressing CFA/I, CS3, CS4/CS6, CS5/CS6 or CS6, or recombinant *E. coli* strains expressing CS1 or CS2 (Table 1) pre-treated with 4% mannose were incubated with 15 µl pooled mouse serum from each group, at room temperature for 30 minutes with shaking (50 rpm). Incubated bacteria were added to confluent monolayer Caco-2 cells. After incubation for one, two, or four hours in a 5% CO_2_ incubator at 37°C, Caco-2 cells were gently washed with PBS to remove non-adherent bacteria and dislodged with 0.5% Triton X-100. *E. coli* bacteria adherent to Caco-2 cells were collected, serially diluted, and plated on LB agar plates. Bacteria grown overnight were counted for CFUs.

**Table 1.** A list of enterotoxigenic *Escherichia coli* field isolates and recombinant *E. coli* strains used for antibody adherence inhibition assays in this study.

Strain	Relevant characteristics	Source
H10407	O78:H11; CFA/I, LT, STa	Johns Hopkins University
THK38/pEU405	CS1	Emory University
DH5/pEU588	CS2	Emory University
E116 (E19446)	CS3, LT, STa	University of Gothenburg
E106 (E11881/9)	CS4/CS6, LT, STa	University of Gothenburg
UM 75688	CS5/CS6, LT, STa	Johns Hopkins University
JF2423 ETP98066	CS6, LT, STa	Washington University

### Statistical analysis

Mouse IgG antibody titration, antibody neutralization activities against bacterial adherence and CT enterotoxicity data were analyzed using a standard one-way ANOVA. A calculated *p*-value less than 0.05 indicated a significant difference.

## Results

### The dmLT adjuvant upregulated the anti-adhesin immune responses to the CFA/I/II/IV vaccine candidate in SC immunized mice

**Figure 1.**
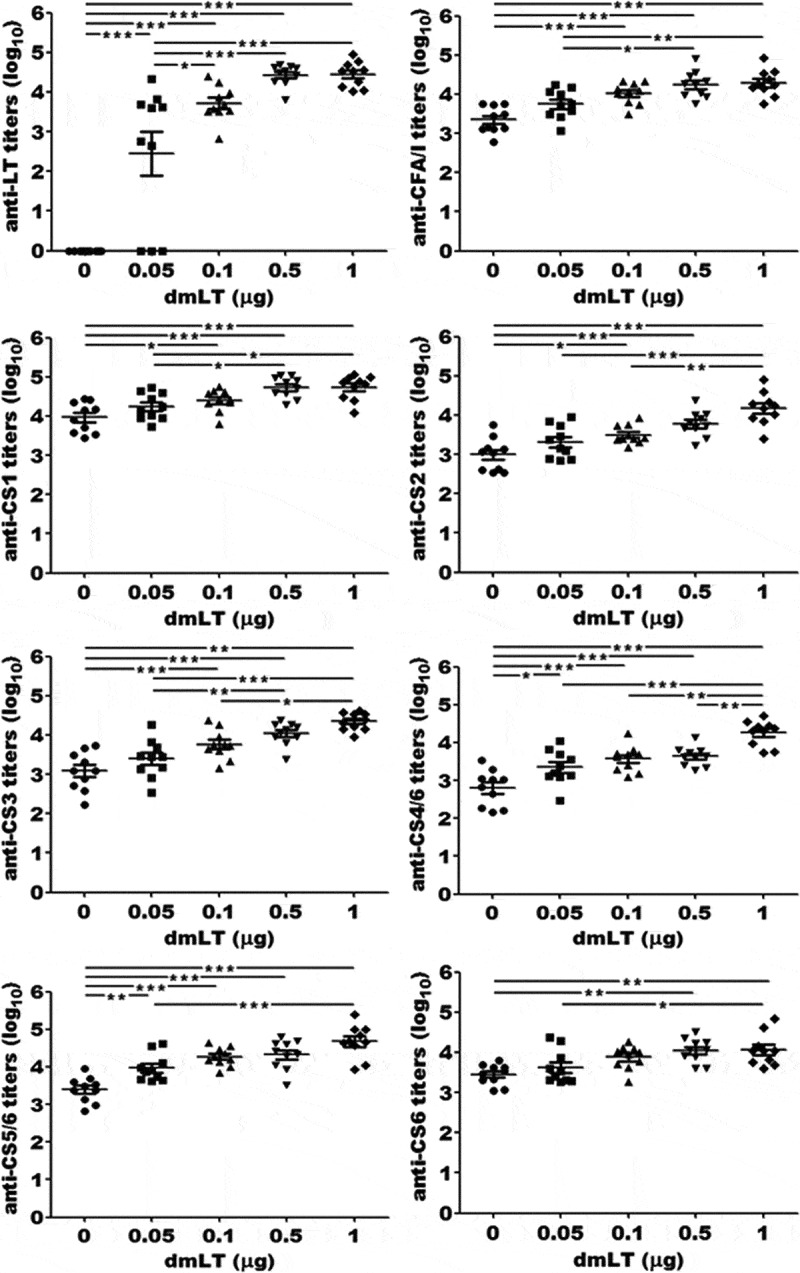
Anti-LT, -CFA/I, -CS1, -CS2, -CS3, CS4, CS5 and anti-CS6 IgG antibody titers (log_10_) in the serum samples of mice immunized with CFA/I/II/IV MEFA with or without dmLT adjuvant. Mice (n = 10) in each group were SC immunized with 16 µg CFA/I/II/IV MEFA and 0, 0.05, 0.1, 0.5 or 1 µg of dmLT. Bars in each group represent the means and standard deviations of IgG titers. Each dot indicates the antibody titer of a mouse. *, **, and *** represent *p*-value of <0.05, <0.01, and <0.001, respectively.

Mice SC immunized with CFA/I/II/IV MEFA with 1 µg dmLT had significantly greater anti-adhesin IgG titers detected in serum samples, compared to mice immunized with CFA/I/II/IV MEFA without dmLT adjuvant (*p* < .01) (Figure 1). Anti-CFA/I, -CS1, -CS2, -CS3, -CS4, -CS5 and anti-CS6 IgG titers were detected at 4.3 ± 0.33, 4.7 ± 0.31, 4.2 ± 0.42, 4.4 ± 0.22, 4.3 ± 0.33, 4.7 ± 0.44, and 4.1 ± 0.40 (log_10_), respectively, in the serum samples of mice SC immunized with CFA/I/II/IV MEFA adjuvanted with 1 µg of dmLT. The IgG titers to each adhesin in mice immunized with the same antigen but no dmLT were 3.4 ± 0.34, 3.4 ± 0.39, 3.0 ± 0.42, 3.1 ± 0.49, 2.8 ± 0.47, 3.4 ± 0.34, and 3.5 ± 0.26 (log_10_), respectively. No antigen-specific IgG antibodies were detected in serum samples of the control group or the serum samples collected prior to immunization of the three study groups.

### The dmLT adjuvant exhibited a dose-dependent effect on serum IgG titers to colonization factor antigens in the MEFA vaccine candidate

Mice immunized with CFA/I/II/IV MEFA and increasing doses of dmLT adjuvant had greater IgG antibody titers specific to CFA/I, CS1, CS2, CS3, CS4, CS5, and CS6 adhesin detected in serum samples (Figure 1). After the dmLT dose reached 0.1 µg, adjuvant dose-dependent enhancement of the anti-adhesin IgG titers were only seen to CS2, CS3 and CS4 IgG titers; whereas increases in anti-CFA/I, -CS1, -CS5 and anti-CS6 IgG titers were no longer detected. IgG titers to these antigens were no longer significantly different between mouse groups given the MEFA vaccine with 0.1 µg to 1 µg of dmLT (Figure 1).

### SC administered dmLT adjuvant induced dose-dependent anti-LT IgG antibodies in mice

Mice immunized with dmLT adjuvant at different doses developed dose-dependent anti-LT IgG antibodies (Figure 1). Anti-LT IgG titers were 2.5 ± 1.8, 3.7 ± 0.44, 4.4 ± 0.27, and 4.5 ± 0.32 (log_10_) in the mice immunized with CFA/I/II/IV MEFA and 0.05, 0.1, 0.5 or 1.0 dmLT adjuvant. No anti-LT IgG was detected in the serum of the group immunized with the antigen but without dmLT adjuvant or the control mice.

Compared to the group immunized with the MEFA vaccine and 0.05 µg of dmLT, anti-LT IgG titers were significantly greater in the groups immunized with dmLT at 0.1 µg (*p* < .05), 0.5 µg (*p* < .001) or 1 µg (*p* < .001) dmLT adjuvant. However, anti-LT titers from the group adjuvanted with 0.1 µg dmLT were not significantly different from the group immunized with 0.5 µg or 1 µg dmLT.

### Serum samples from mice immunized with MEFA + dmLT neutralized CT enterotoxicity in vitro

**Figure 2.**
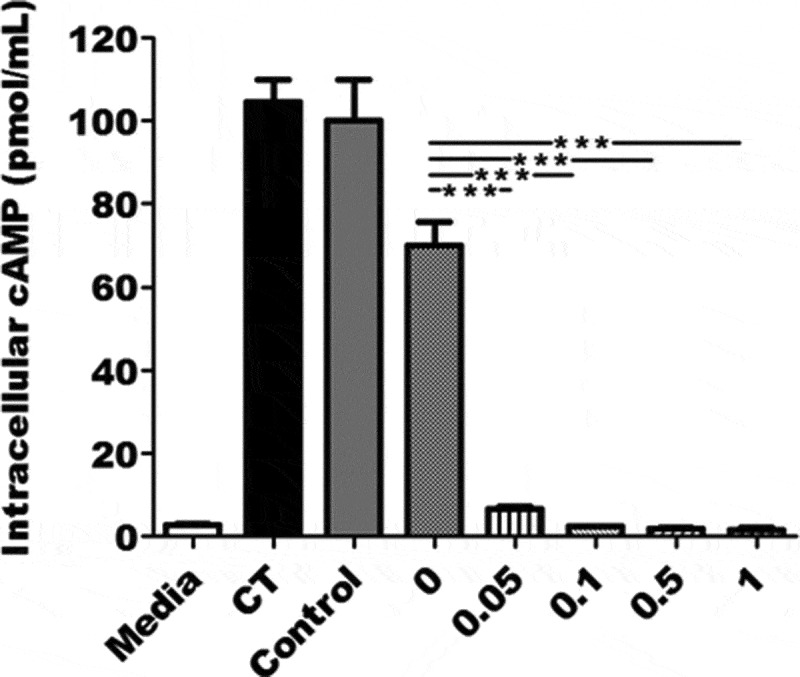
Mouse serum antibody neutralization activity against cholera toxin (CT). Serum samples pooled from each immunization group (n = 10) or the control group mixed with 10 ng CT toxin were added to T-84 cells. Cells were lysed after 3 h incubation, and cell lysates were measured for intracellular cAMP levels by using cAMP EIA kit (Enzo Life Sciences). The mean and standard deviation of each group represented as columns and bars. *** indicates a *p*-value of <0.001.

Mouse serum antibodies in the immunized groups with dmLT adjuvant showed *in vitro* neutralizing activity against CT enterotoxicity (Figure 2). Moreover, dmLT adjuvant doses correlated with antibody neutralizing activity levels against CT. The intracellular cAMP levels in T-84 cells exposed to CT and the serum samples from the immunized mice using 0.05, 0.1, 0.5 or 1.0 µg dmLT adjuvant were 6.6 ± 0.91, 2.3 ± 0.04, 1.8 ± 0.55, and 1.7 ± 0.54 (pmole/ml), respectively. These cAMP levels were significantly different from the level in cells exposed to the CT and the serum from mice immunized without dmLT adjuvant (70 ± 7.8 pmole/ml; *p* < .001).

### Mouse serum antibodies inhibited adherence of ETEC or recombinant E. coli bacteria expressing CFA/I, CFA/II, or CFA/VI antigens

**Figure 3.**
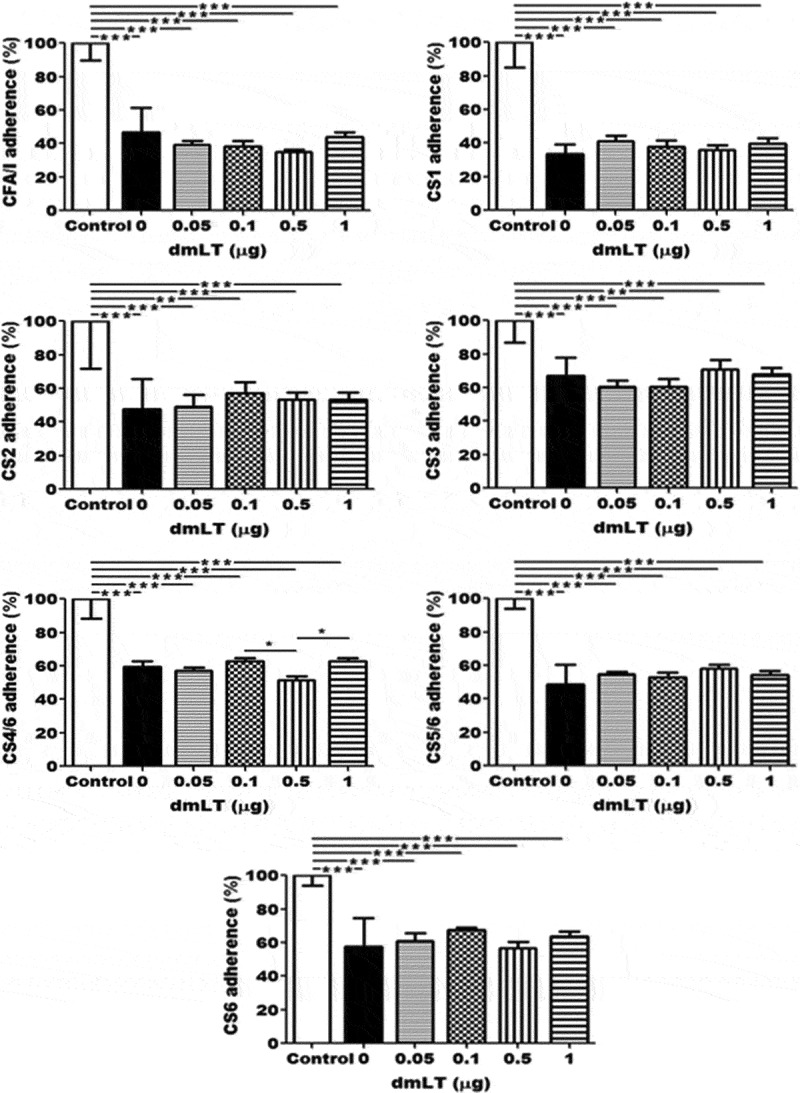
Mouse serum antibody adherence inhibition activity against ETEC or *E. coli* bacteria expressing CFA/I, CS1-CS6 adhesins. ETEC or recombinant *E. coli* expressing CFA/I, CS1, CS2, CS3, CS4/CS6, CS5/CS6, or CS6 adhesin (Table 1), after incubated with mouse serum samples pooled from each immunization group (n = 10) or the control group, were transferred to Caco-2 cells. Incubated for 1 h, cells were washed to remove non-adherent bacteria and lysed. Adherent bacteria were collected, diluted, and plated on LB agar plates. Bacteria were counted for CFUs after overnight growth at 37 ◦C. The number of adherent bacteria in the control group was referred as 100%. ** and *** indicate *p*-value of <0.01 and <0.001, respectively.

Mouse serum samples pooled from the groups SC immunized with CFA/I/II/IV MEFA showed significant inhibition activities against adherence of ETEC bacteria expressing CFA/I, CS3, CS4/CS6, CS5/CS6, or CS6 and recombinant *E. coli* expressing CS1 or CS2 to Caco-2 cells, compared to the serum samples from the control group (Figure 3). Differences on inhibition activity between groups immunized with CFA/I/II/IV MEFA plus 0, 0.05, 0.1, 0.5 or 1.0 µg of dmLT adjuvant were no statistically significant. Additionally, a reduction in mouse serum volume from 15 µl to 7.5 µl or increases of incubation time from one hour to two hours or four hours showed the same outcomes of antibody adherence inhibition assays.

## Discussion

Results from this study demonstrate that dmLT is an effective adjuvant for upregulating immune responses to the SC administered ETEC CFA/I/II/IV MEFA vaccine candidate for anti-adhesin antibody responses. The dmLT adjuvant has been applied recently in intraperitoneal (IP) or intramuscular (IM) immunization with ETEC antigens in mice or pigs, but adjuvanticity of dmLT was largely not specifically characterized in those studies.^22,24,28,29^ One study directly compared dmLT with Freund’s adjuvant (Sigma) in the IP route or ISA51 (SEPPIC) adjuvant in the SC route, and results indicated that the dmLT was equally effective as ISA51 or better than Freund’s adjuvant in immunoregulating ETEC toxoid fusion antigen 3xSTa_N12S_-mnLT_R192G/L211A_ (previously named as 3xSTa_N12S_-dmLT) for antibodies to two ETEC toxins, LT and STa.^22^ However, since dmLT adjuvant induces anti-LT antibody response, as demonstrated by the current study (Figure 1), dmLT adjuvanticity for the ST-LT toxoid fusion was inconclusive, since anti-LT antibodies were not significantly enhanced. Data from this study showed mice immunized with the CFA/I/II/IV MEFA, which has no antigenic homology with dmLT, in the presence of dmLT adjuvant developed significantly greater IgG antibody responses to each of the seven target adhesins, compared to the mice immunized with the same antigen but no dmLT adjuvant, thus indicating dmLT adjuvant activity. Similar results were observed in a previous study.^25^ While anti-CFA/I to CS6 IgG antibody titers (log _10_) from the mice IP immunized with CFA/I/II/IV MEFA and Freund’s complete adjuvant varied from 1.3 ± 1.0 to 3.5 ± 0.1, the titers from mice SC injected with CFA/I/II/IV MEFA and 0.1 µg of dmLT in this study ranged from 3.5 ± 0.2 to 4.4 ± 0.2, suggesting the superior adjuvanticity of dmLT.^25^

This study demonstrated a positive effect of dmLT adjuvant upon CFA/I/II/IV MEFA immunogenicity in a dose-dependent way. Since antibody enhancement plateaued at a SC dose of 0.1 µg of dmLT, 0.1 µg dmLT is suggested a preferable adjuvant dose for SC immunizing mice with CFA/I/II/IV MEFA. In an early study that used two doses of dmLT adjuvant (0.2 µg and 2.0 µg) given with a new LT-ST toxoid fusion, a dose-dependent effect on the anti-toxoid antibody was not observed.^22^ Antibody responses to LT were improved but no enhancement of the antibody response to ST was seen. The increased anti-LT antibody response appears to be directly attributable to dmLT anti-LT antigenicity rather than adjuvanticity. Nevertheless, dmLT enhancement of anti-LT antibody response is a desirable outcome, since LT is an important virulence factor for ETEC, which is a major cause of both travelers’ diarrhea, as well as acute diarrhea and stunting among young children and infants living in high-risk areas.

The current study included five doses of dmLT adjuvant (0 µg, 0.05 µg, 0.1 µg, 0.5 µg and 1.0 µg) and comparable antibody enhancement was observed at the 0.1, 0.5 and 1 µg doses. These observed better adjuvant doses are comparable to the doses currently under evaluation in Phase 1 trials of dmLT as a prototype ETEC vaccine antigen (DMID Protocol 12–0023, NCT02052934; DMID Protocol 13–0013, NCT02531685; Bernstein et al., Vaccine, 37(4):602–11, 2019), including a study examining the impact of adding dmLT to a prototype ETEC subunit antigen given by the IM route (PATH Protocol VAC 050, NCT03404674). Consequently, the mouse studies detailed in this report suggest that the dmLT doses being studied in the human clinical studies noted above would be similarly beneficial for the MEFA vaccine candidate and help to set the stage for moving the MEFA + dmLT combination into Phase 1 human clinical trials. However, the concluded dosage effect of dmLT adjuvant was based on the CFA/I/II/IV MEFA antigen in mouse subcutaneous immunization. In addition, since that CFA/I/II/IV MEFA and toxoid fusion 3xSTa_N12S_-mnLT_R192G/L211A_ are currently targeted by Zhang laboratory for developing an ETEC subunit vaccine, it would be programmatically beneficial to examine dmLT adjuvant dose effect at up-immunoregulation of toxoid fusion antigen in the conventional IM route or the novel intradermal route using the microneedle patch approach.^24^

Additionally, human volunteer studies will be needed to identify the optimal dose combination for the dmLT adjuvant and the MEFA and LT-ST fusion toxoid since an optimal dose for animals may not be directly extrapolated to humans. While a clear dmLT dose-dependent immune-enhancing effect on anti-adhesin IgG antibodies titers to the CFA/I/II/IV MEFA was shown, correlation of dmLT adjuvant doses and antibody activities in inhibiting ETEC or *E. coli* bacterial *in vitro* adherence was not observed from the current study. This could be caused by the high titers of antibodies to CFA/I and CS1 to CS6 in mice serum derived from different doses of dmLT or even no dmLT, resulting in difficult to discriminate the inhibition activities among groups. Further assessment with adjusting the incubation time and/or at different ratios of ETEC bacteria and serum samples might be needed to detect dose-dependent inhibition activity. We should also point out that the result from this study requires *in vivo* protection verification. Future studies using a rabbit colonization model or human subject field trials will hopefully determine the dose effect of dmLT adjuvant on the functionality and protective efficacy of the improved anti-adhesin antibody responses to this MEFA candidate vaccine against ETEC colonization and disease. Furthermore, it is worthwhile to investigate cell-mediated immune responses including cytokine changes following different doses of dmLT to optimize the dose of dmLT adjuvant as well as candidate vaccine in parenteral route. Because of low and variable responses at the initial analyses, anti-adhesin or anti-LT IgA data were excluded from this study. Future studies to examine the dmLT adjuvant’s effect on regulating antigen-specific IgA, particularly mucosal IgA antibody responses and the correlation of local IgA responses to disease prevention, are also needed.
